# Supplementary material for: Understanding the impact of fall armyworm (Spodoptera frugiperda J. E. Smith) leaf damage on maize yields
Source: PLoS One. 2023 Jun 12;18(6):e0279138. doi: 10.1371/journal.pone.0279138 (PMC10259777; doi:10.1371/journal.pone.0279138)

**Supplementary materials Figure S1**

**Figure S1** Cumulative leaf damage against the cumulative number of weeks exposure to fall armyworm larval feeding. According to different treatments, plants were innocualted with 2^nd^ instar fall armyworm larvae at 2, 4 and 6 weeks after seedling emergence, and some larvae were removed after 1 week while others were allowed to feed for 2 weeks, which was sufficient to reach the 6^th^ instar or pupae stages. Larvae sometimes migrated to neighbouring plants, hence the data shown are the observed weeks of exposure to larval feeding. Leaf damage was assessed at 3, 5 and 7 weeks after seedling emergence using the Davis 9 point scale, which was rescale from 0-8. A) Early maturing variety; B) Medium maturing variety; C) Late maturing variety.

1A Early maturing


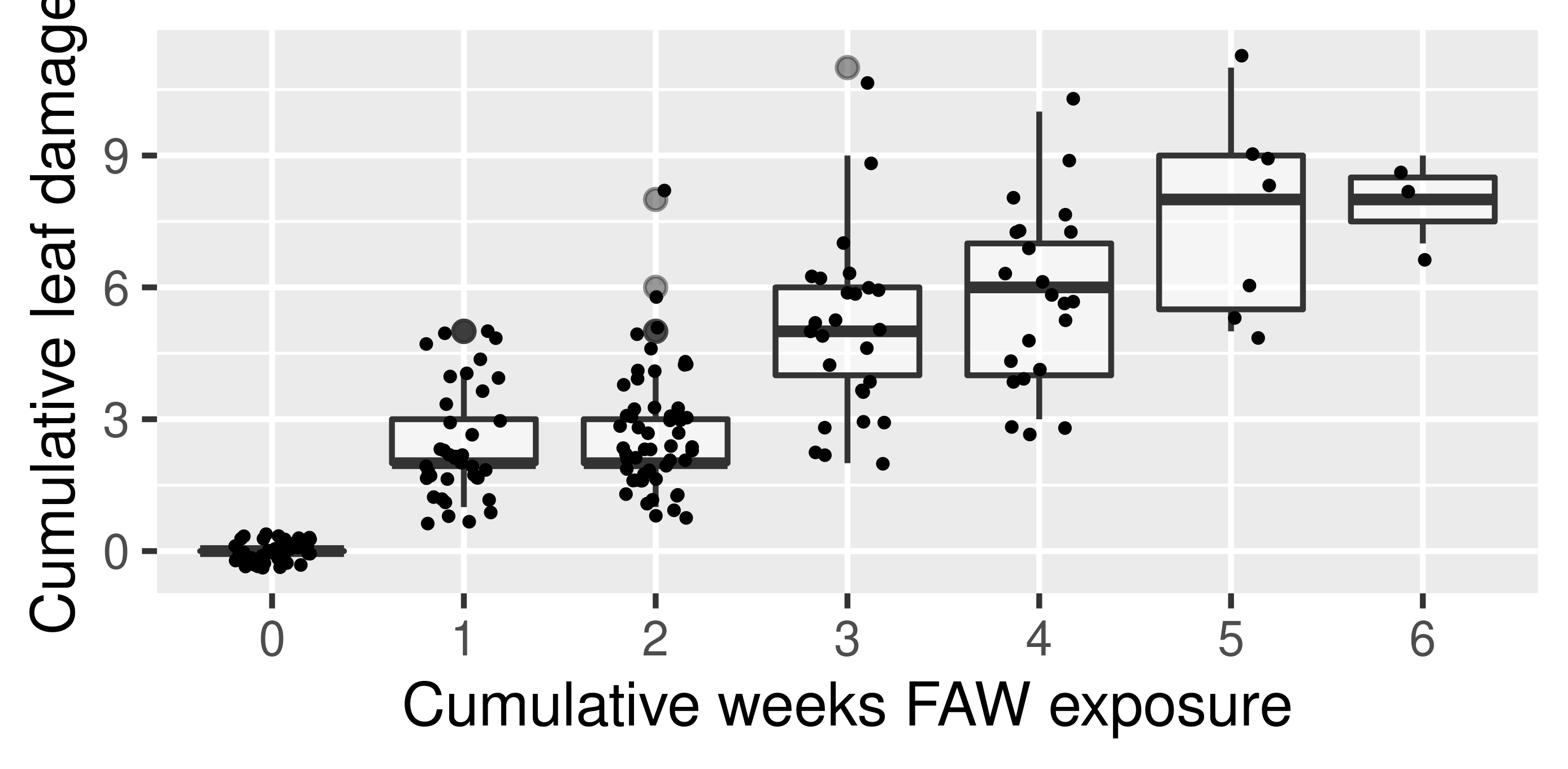


1B Medium maturing


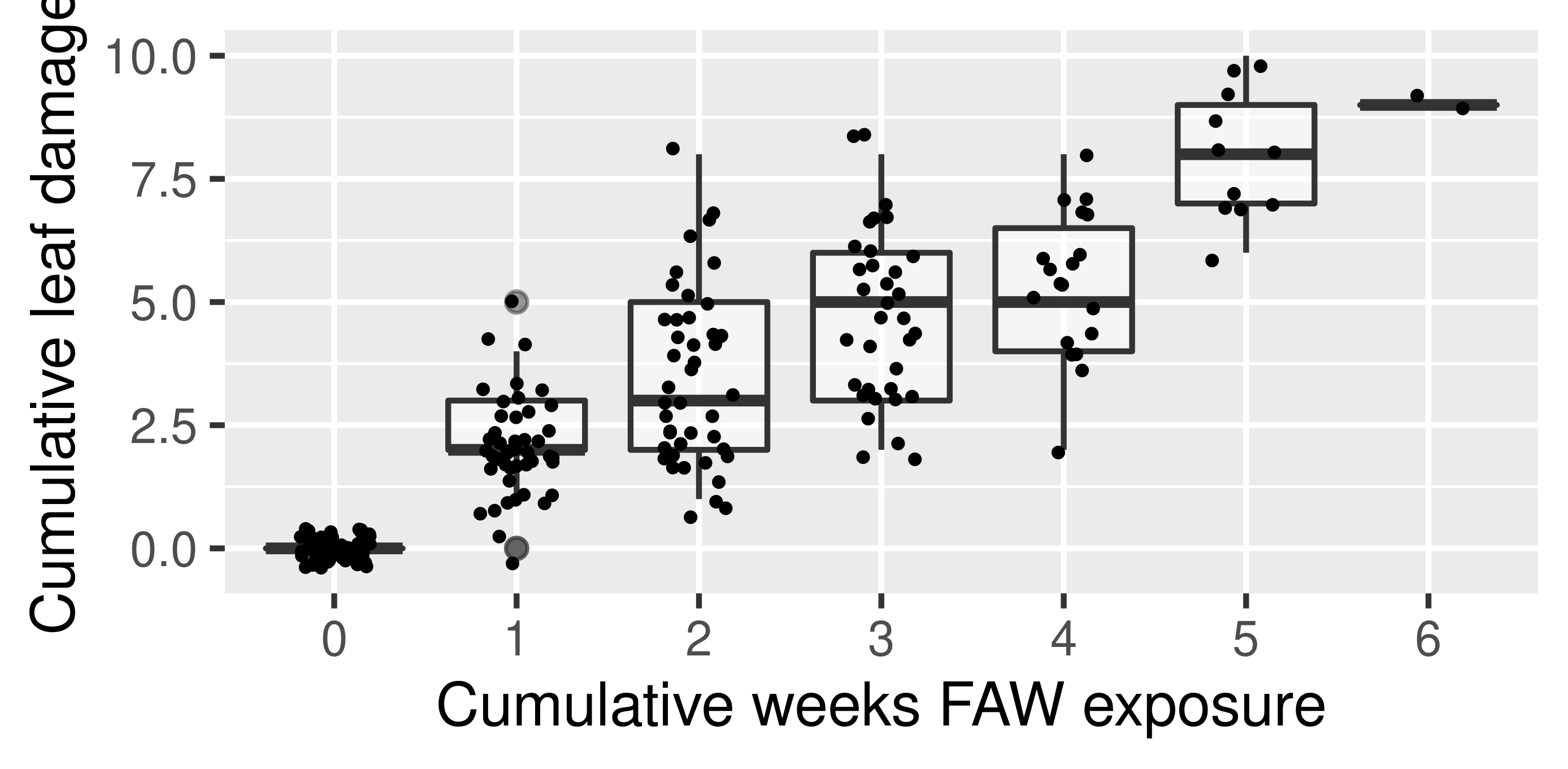


1C Late maturing


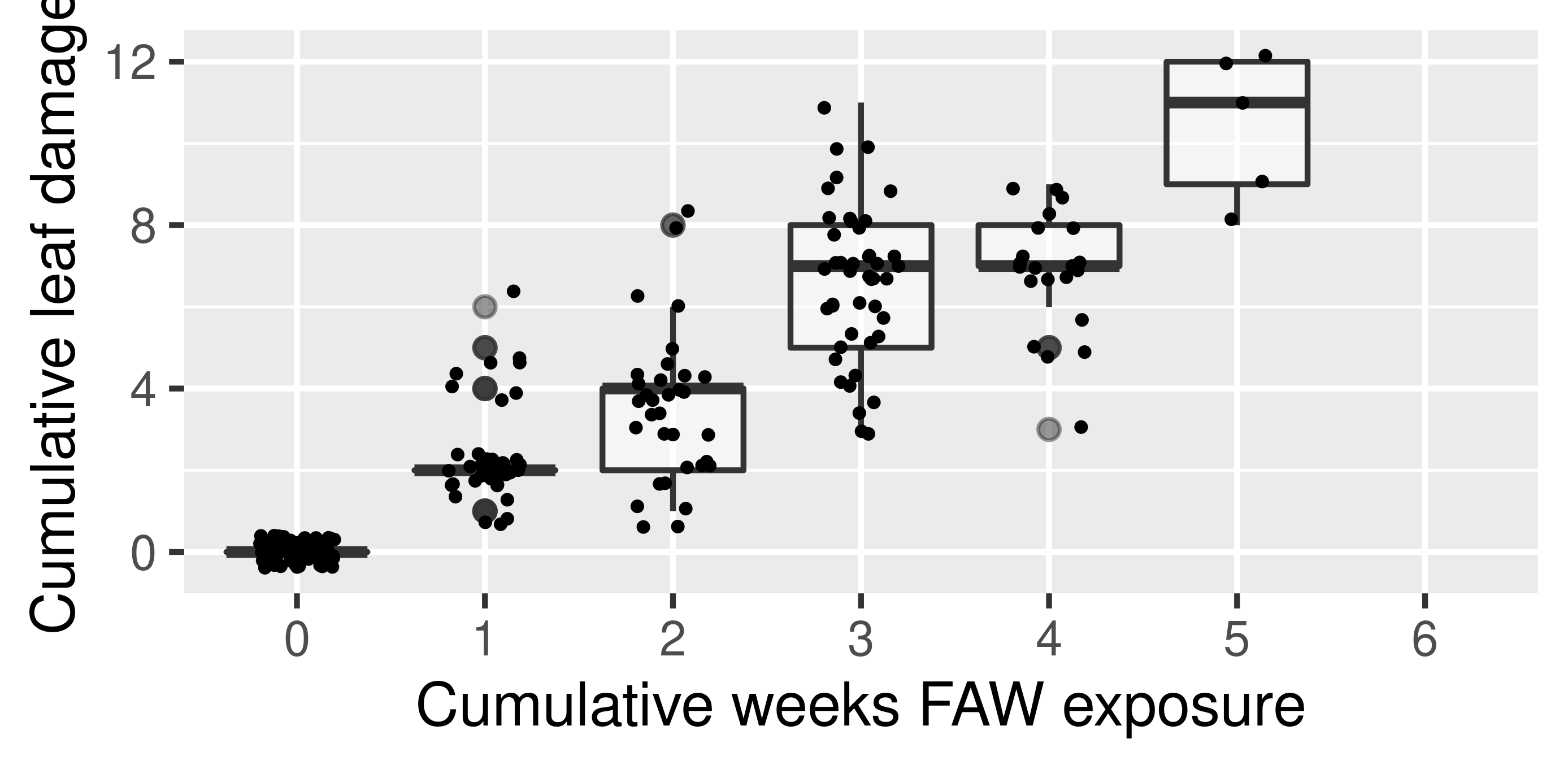

Supplement: S1 Fig — According to different treatments, plants were innocualted with 2nd instar fall armyworm larvae at 2, 4 and 6 weeks after seedling emergence, and some larvae were removed after 1 week while others were allowed to feed for 2 weeks, which was sufficient to reach the 6th instar or pupae stages. Larvae sometimes migrated to neighbouring plants, hence the data shown are the observed weeks of exposure to larval feeding. Leaf damage was assessed at 3, 5 and 7 weeks after seedling emergence using the Davis 9 point scale, which was rescale from 0–8. A) Early maturing variety; B) Medium maturing variety; C) Late maturing variety. (DOCX) [file pone.0279138.s001.docx]
