# Supplementary material for: Understanding the impact of fall armyworm (Spodoptera frugiperda J. E. Smith) leaf damage on maize yields
Source: PLoS One. 2023 Jun 12;18(6):e0279138. doi: 10.1371/journal.pone.0279138 (PMC10259777; doi:10.1371/journal.pone.0279138)
Supplement: S1 Table — The V5, V8, V12, VT and R1 indicate the maize stage. L = Larval inoculation. RM indicates larvae were removed after 7 days. (DOCX) [file pone.0279138.s002.docx]

**Supplementary materials Table S1**

Table S1: Summary of treatments. The V5, V8, V12, VT and R1 indicate the maize stage. L = Larval inoculation. RM indicates larvae were removed after 7 days.

| **Treatment Code** | **FAW inoculation** |
| --- | --- |
| A | Control |
| B | L@V5 |
| C | L@V5 + RM |
| D | L@V8 |
| E | L@V8 + RM |
| F | L@12 |
| G | L@12 + RM |
| H | L@VT |
| I | L@VT + RM |
| J | L@R1 |
| K | L@R1 + RM |
| L | B + D |
| M | C + D |
| N | B + E |
| O | C + E |
| P | D + F |
| Q | E + F |
| R | D + G |
| S | E + G |
| T | B + D + F |
| U | C + D + F |
| V | B + E + F |
| W | B + D + G |
| X | C + E + F |
| Y | C + D + G |
| Z | B + E + G |
| AA | C + E + G |
